# The Roles of Base Modifications in Kidney Cancer

**DOI:** 10.3389/fonc.2020.580018

**Published:** 2020-11-13

**Authors:** Chunyue Feng, Xiaoli Huang, Xuekun Li, Jianhua Mao

**Affiliations:** ^1^ The Children’s Hospital, Zhejiang University School of Medicine, Hangzhou, China; ^2^ National Clinical Research Center for Child Health, Hangzhou, China; ^3^ Institute of Translational Medicine of Zhejiang University School of Medicine, Hangzhou, China

**Keywords:** DNA methylation, DNA hydroxymethylation, RNA methylation, ten-eleven translocases, tumor

## Abstract

Epigenetic modifications including histone modifications and DNA and RNA modifications are involved in multiple biological processes and human diseases. One disease, kidney cancer, includes a common type of tumor, accounts for about 2% of all cancers, and usually has poor prognosis. The molecular mechanisms and therapeutic strategy of kidney cancer are still under intensive study. Understanding the roles of epigenetic modifications and underlying mechanisms in kidney cancer is critical to its diagnosis and clinical therapy. Recently, the function of DNA and RNA modifications has been uncovered in kidney tumor. In the present review, we summarize recent findings about the roles of epigenetic modifications (particularly DNA and RNA modifications) in the incidence, progression, and metastasis of kidney cancer, especially the renal cell carcinomas.

## Introduction

Kidney cancer presents about 2% of all cancers and is the seventh most common cancer worldwide with 295,000 new cases being diagnosed annually (1). The most prevalent solid tumor of the kidney in adults is renal cell carcinoma (RCC), which accounts for about 90% of adult kidney cancer (2–4). RCC is a heterogeneous malignant tumor with more than ten histological subtypes, although it mainly stems from renal tubular epithelial cells. In addition to the high prevalence of kidney cancer in adults, this disease can also be diagnosed in children, where the main form is Wilms tumor (5). Because of the high malignancy rate and the unclear mechanisms of kidney cancer, current treatments, which include surgery, chemotherapy and radiation, cannot significantly inhibit tumor progression. In the past few years, targeted therapy has been shown to prolong survival of patients, but the overall survival rate still remains very low (4).

Epigenetic modifications including histone modifications, DNA and RNA modifications, and non-coding RNAs regulate gene expression at transcriptional, translational and posttranslational levels and therefore are involved in human diseases (6). DNA methylation at the 5’ position of cytosine (5-methylcytosine, 5mC) is an intensively studied type of epigenetic modification, and it plays a critical role in development and diseases (7). In addition, more than one hundred types of RNA modifications have been identified on mRNA, tRNA, etc. Among all RNA modifications, *N* ^6^-methyladenosine (m^6^A) is the most common modification in eukaryotic mRNAs (8). RNA modification has been shown to play important roles in multiple biological processes and in diseases, as well as in DNA methylation (9). The dysfunction of epigenetic modifications leads to global changes in genomic structure and thus affects the expression of genes involved in cancer progression (10, 11).

During the past decade the important roles of epigenetic modifications have been revealed in kidney cancer (especially in RCC). Epigenetic alterations have been suggested as promising biomarkers for RCC diagnosis and potential therapeutic targets (3, 4, 11–14). In this review we summarize the landscape of main epigenetic modifications with a focus on DNA methylation and RNA methylation. We then discuss the function and underlying mechanisms of aberrant DNA and RNA modifications in kidney cancer.

### DNA Modifications and Kidney Cancer

#### Diverse Modifications of DNA

**Figure 1:**
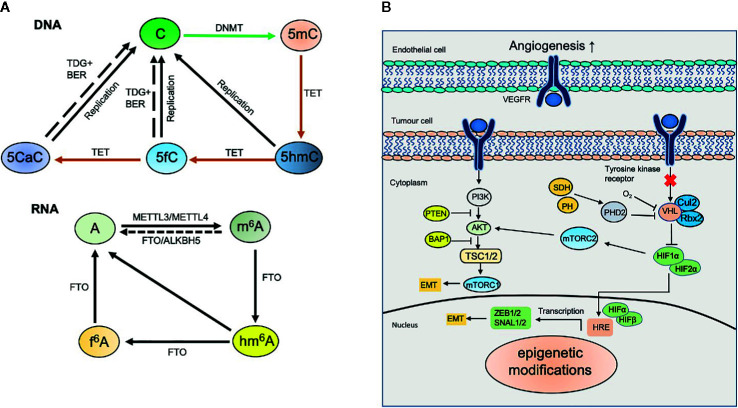
Dynamic modifications in kidney cancer. **(A)** Dynamic DNA and RNA modifications. DNA methyltransferases (DNMTs) including *de novo* methyltransferases DNMT3A, DNMT3B and maintenance methyltransferase DNMT1 convert unmodified cytosine (C) to 5‑methylcytosine (5mC). 5mC can be converted to 5‑hydroxymethylcytosine (5hmC) by ten-eleven translocation (TET) proteins‑mediated oxidation. TET proteins also catalyze the oxidation of 5hmC to 5‑formylcytosine (5fC) and 5‑carboxylcytosine (5caC). 5fC and 5caC can be further excised by thymine DNA glycosylase (TDG) coupled with base excision repair (BER) to generate unmodified cytosine. *N^6^*-methyladenosine (m^6^A) in mRNA is installed by methyltransferase-like protein 3 (METTL3) and METTL14, and erased by fat mass and obesity-associated protein (FTO) and α‑ketoglutarate-dependent dioxygenase alkB homologue 5 (ALKBH5). m^6^A can be further oxidized to *N^6^*-hydroxymethyladenosine (hm^6^A) and *N^6^*-formyladenosine (f^6^A) sequentially by FTO. **(B)** Epigenetic modifications involve in kidney cancer. Epigenetic modifications regulate diverse signaling pathways including HIF and PI3K-AKT and involve in kidney cancer.

DNA methylation mainly occurs at the fifth carbon atom of cytosine (5mC) in mammalian DNA and is catalyzed by DNA methyltransferases (DNMTs), which use S-adenosyl methionine (SAM) as a methyl group donor. Currently, there are five members of the DNMT family, which includes DNMT1, DNMT3a, and DNMT3b. DNMT1 displays a preference for hemi-methylated DNA at the CpG islands during DNA replication, whereas DNMT3a and DNMT3b are *de novo* methyltransferases. DNA methylation exhibits dynamic features of expression during embryonic and postnatal development, and the dysregulation of DNA methylation has been shown to result in changes in gene expression (15). In general, hypomethylation activates or increases gene expression, whereas hypermethylation leads to gene silencing or decreased gene expression (**Figure 1**).

For quite some time, 5mC has been considered as a stable epigenetic marker of DNA that cannot be further modified. However, in 2009 researchers found that 5mC can be oxidized to 5-hydroxymethylcytosine (5hmC) under the catalysis of ten-eleven-translocation (TET) family proteins (16–18). The TET family proteins consist of three members, TET1, TET2, and TET3, which share common typical characteristics of 2-oxoglutarate (2OG)- and Fell(II)-dependent dioxygenases (2OGFeDO) (19–21). TET enzymes can further oxidize 5-hmC to 5-carboxylcytosine (5caC) and 5-formylcytosine (5fC) (22, 23). Thymine-DNA glycosylase (TDG) is in charge of recognition and excision of 5caC and 5fC in mammals (22, 23). Under the catalysis of activation-induced cytidine deaminase (AID), 5mC can be transformed to 5-hydroxymethyluracil (5hmU) with a deamination reaction. In addition, previous studies have demonstrated that IDH1/2 can catalyze isocitrate to α-KG and can participate in the regulation of TETs and 5hmC (24). However, mutant IDH1/2 has been found to catalyze isocitrate to 2-hydroxyglutarate (2-HG), which is a competitive inhibitor of α-KG. IDH1/2 also can inhibit the transformation of 5mC to 5hmC by TETs; therefore, it plays a pivotal role in the regulation of 5hmC (24).

Current findings indicate that 5-hmC modification not only serves as an intermediate product, but also plays a pivotal role in development, aging, and diseases. Tissue/cell-specific distribution features and the content of 5hmC have been observed among varied tissues and organs. 5hmC is the most abundant in neuronal cells compared to other types of cells. In addition, 5hmC is mainly enriched at gene bodies, promoters, and distal regulatory regions of the genome. The enrichment of 5hmC at distinct genomic regions is correlated with gene expression, which can also be regulated by histone modifications (25). Abnormal distribution and/or level of 5hmC modification can induce disease. All of these findings suggest several important functions for dynamic DNA modifications.

#### The Function of DNA Methylation in Kidney Cancer

The aberrant level and distribution of DNA methylation have been revealed in various types of cancer including liver, colon, lung, and prostate cancer. These cancers are associated with the severity and metastatic potential of diseases (26). For example, DNA hypermethylation in cancer cells may be an alternative complementary mechanism, which triggers the silence of tumor-inhibiting genes and consequently results in tumorigenesis and metastasis (10, 27). In general, the global level of DNA methylation is decreased, while the acquisition of DNA methylation is observed at the promoter regions of some specific genes.

In studies of kidney cancer, Chen et al. applied the bisulfite sequencing method to map 5mC and found that the global level of 5mC is not changed (28). However, Mendoza-Pérez J et al. performed the analysis of 899 RCC cases and found that a low level of genomic DNA methylation (measured as 5mC%) in peripheral blood could significantly increase the risk of RCC (29). One possibility for these inconsistent results could be the ability of the methods used to distinguish DNA methylation and demethylation.

#### The Function of DNA Demethylation in Kidney Cancer

Mounting evidence has demonstrated that 5hmC plays an important function in a variety of tumors, such as acute myeloid leukemia, liver cancer, and melanoma (30, 31). Although the level of global 5mC is not altered, Chen et al. observed the decreased level of global 5hmC as well as the hypermethylation at gene body regions in kidney tumors (28). Their results also suggested that decreased 5hmC is correlated with the prognosis and survival. It has also been found that 5hmC is closely related with capsule invasion, vein invasion and clinical progress of RCC (32). RCC patients with high level of 5hmC show increased survival; therefore, 5hmC may serve as an independent prognostic and progression marker for RCC (32). Consistently, 5hmC hydroxymethylase TET1 can promote cell apoptosis and can inhibit cell proliferation and invasion, therefore inhibiting tumor growth in RCC (33). The inhibited expression of TET1 reduces 5hmC level at the promoter region of CCNY/CDK16 and consequently results in cell cycle arrest and inhibits self-renewal of renal cancer stem cells (34) (**Figure 1**).

The oxidation reaction of 5mC to 5hmC requires 2-ketoglutarate (2-KG) as co-substrates, which is generated by isocitrate dehydrogenase 1 (IDHs) during the tricarboxylic acid cycle (TCA). The down-regulated expression of IDH1 in kidney cancer contributes to the global loss of 5hmC in RCC (28). Consistently, ectopic expression of IDH1 and pharmacologically increasing intracellular 2-KG can restore the global levels of 5hmC, and consequently, can inhibit tumor growth (28, 35). IDH1 mutation leads to the increase of 2-hydroxyglutarate (2-HG), and the loss of 5hmC is partly mediated by the decrease 2-HG dehydrogenase (L2-HGDH), which has tumor inhibitory effects (36). The loss of L2HGDH is correlated with a worse prognosis, whereas the restoration of L2HGDH can increase 2-HG and can promote the accumulation of 5hmC in RCC cells (37). Ascorbic acid (AA), a cofactor for TET, can enhance the activities of TET enzymes and can restore the level of genomic 5hmC, thus reversing epigenetic aberrancy (38, 39). These findings suggest an interplay between DNA demethylation and metabolites that has an important role in kidney cancer (39, 40).

## RNA Methylation and Kidney Cancer

### Diverse Modifications of RNA and Molecular Mechanism of m^6^A Modification

To date, more than 110 types of RNA modifications have been identified, such as *N^1^*-methyladenosine (m^1^A), *N^6^*-methyladenosine (m^6^A), *N^6^*-methyl-2′-O-methyladenosine (m^6^A_m_), 5-methylcytosine (m^5^C), 5-hydroxymethylcytosine (hm^5^C) in messenger RNA (mRNA), transfer RNA (tRNA), ribosomal RNA (rRNA), long non-coding RNAs (lncRNAs), etc (41). Among these modifications, m^6^A is the most abundant internal chemical modification in eukaryotic mRNA. In mammals, 0.1%–0.4% of adenosines (~3–5 m^6^A sites per mRNA) are modified by m^6^A, accounting for nearly half of total methylated ribonucleotides (42). m^6^A mainly enriches at the 3′ untranslated regions (3′UTRs), around the termination codons and the internal long exons (43).

m^6^A modification is mediated by three key elements called “writers”, “erasers”, and “readers” (44, 45). m^6^A modification is mainly catalyzed by the RNA methyltransferase complex (writers), including methyltransferase-like 3 and 4 (METTL3 and METTL14) and Wilms’ tumor 1-associated protein (WTAP) (46). METTL3 is in charge of m^6^A installation, while METTL14 participates in the interacting with target mRNA, and WTAP is responsible for the localization in the nuclear speckle (47). m^6^A modifications can be removed by RNA demethylases (erasers), including alkB homolog 5 (ALKBH5) and fat mass and obesity-associated protein (FTO, alpha-ketoglutarate dependent dioxygenase) (48). Both ALKBH5 and FTO belong to the alpha-ketoglutarate dependent dioxygenase family, which catalyze m^6^A demethylation in a Fe(II)-and alpha-ketoglutarate dependent manner. Similar to ALKBH5, alkB homolog 3 (ALKBH3) has been shown the demethylase activity for 1-methyladenine and 5-methylcytosine (49). m^6^A readers include the YTH domain family (YTHDF), insulin-like growth factor 2 mRNA binding protein 2 (IGF2BP), and HNRNPA2B1 (50). YTHDF proteins act as m^6^A readers, which can maintain the stability of m^6^A transcripts (51, 52) (**Figure 1**).

The dynamic and reversible m^6^A modification regulates various aspects of RNAs fate, such as nuclear exit, splicing, stability, efficiency of translation (41, 53); therefore, this modification has crucial roles in embryonic development, sex determination, neurogenesis, stress responses, and tumorigenesis in mammals (54, 55). Previous studies have shown that the dysregulation of m^6^A was induced, but was not limited to, the aberrant expression of its writers, erasers and readers. These result in profound outcomes in multiple biological processes, such as cell proliferation and fate determination, DNA damage response, embryogenesis, and heat shock responses, and therefore are involved in diseases (56–59). In addition, emerging evidence indicates that m^6^A modification plays a significant role in tumorigenesis and progression of a variety of cancers including breast cancer, gastric cancer, and pancreatic cancer (49, 55, 60–62).

### The Function of m^6^A in Renal Cell Carcinoma

Although the function of m^6^A has been shown in several types of tumors, the important roles of m^6^A in RCC are still not completely known. Recent findings show that the level of global m^6^A decreases in RCC compared with adjacent non-tumor tissues (63), suggesting that the expression of m^6^A regulatory genes may be a biomarker for RCC. The protein level of m^6^A eraser FTO displays a significant decrease in RCC compared with normal tissues (64). Lower levels of m^6^A modification eraser FTO are usually associated with malignant prognosis whereas higher levels of FTO are associated with benign prognosis, suggesting that FTO may serve as a protective factor in RCC (65). Published findings about the role of ALKBH5 in RCC are controversial. Both increased and decreased expression of ALKBH5 in RCC have been reported (64, 66). In a retrospective study using TCGA database, Zhou et al. examined the alteration of m^6^A regulatory genes in clear cell renal cell carcinoma (ccRCC) and found that these m^6^A regulatory genes are significantly correlated with von Hippel-Lindau (*VHL*) and *TP53*, two key suppressors for RCC. This result suggests a relationship between m^6^A regulatory genes and the pathologic stage (63); however, it still lacks solid evidence about the roles of m^6^A writers METTL3 and METTL14 in RCC (**Figure 1**).

In human RCC tissues, mitochondrial enzyme methylenetetrahydrofolate dehydrogenase 2 (MTHFD2) is highly expressed, and the knockdown of *MTHFD2* inhibits cell migration and invasion (67). High level of *MTHFD2* is positively correlated with RCC grade, clinical stage, progression, and poor prognosis (68). Interestingly, *MTHFD2* knockdown leads to a decrease of global m^6^A, and a hypomethylation of HIF-2α mRNA increases the translation of HIF-2α (67, 69), which in turn promotes the aerobic glycolysis (67). These findings establish a connection between m^6^A modification and MTHFD2-mediated one-carbon metabolism in RCC.

## Conclusions

During the past several decades, significant progress has been made in understanding the function of epigenetic modifications in kidney cancer. However, the detailed molecular mechanisms underlying the kidney cancer carcinogenesis are still not completely known, and it has been challenging to explore the accurate diagnosis and effective treatment of kidney cancer. First, the interactions between DNA modifications, RNA modifications, and histone modifications in regulating gene expression in kidney cancer need to be determined. How these interactions cooperate to regulate diverse signaling pathways involved in kidney cancer requires further clarification. Second, the precise map of DNA and RNA modifications should be established in kidney cancer with high-throughput sequencing technologies. The identification of therapeutic targets relies on the analysis of high-throughput sequencing data. The therapeutic implications of epigenetic hallmarks are to be expected in kidney cancer considering the successful application of these hallmarks in other types of cancers.

## Author Contributions

CF, XH, JM, and XL wrote the manuscript. All authors contributed to the article and approved the submitted version,

## Funding

This work was supported in part by the Medicine & Health Technology Project of Zhejiang Province (2018RC007 to CF), Zhejiang Provincial Research Center for Cancer Intelligent Diagnosis and Molecular Technology (JBZX-202003 to JM) and the National Natural Science Foundation of China (grants 31571518, 31771395 to XL). 

## Conflict of Interest

The authors declare that the research was conducted in the absence of any commercial or financial relationships that could be construed as a potential conflict of interest.
